# Monocyte derived macrophages from CF pigs exhibit increased inflammatory responses at birth

**DOI:** 10.1016/j.jcf.2017.03.007

**Published:** 2017-07

**Authors:** Lily Paemka, Brian N. McCullagh, Mahmoud H. Abou Alaiwa, David A. Stoltz, Qian Dong, Christoph O. Randak, Robert D. Gray, Paul B. McCray

**Affiliations:** a Department of Pediatrics, Pappajohn Biomedical Institute, Carver College of Medicine, University of Iowa, Iowa City, IA, USA; b Department of Internal Medicine, Pappajohn Biomedical Institute, Carver College of Medicine, University of Iowa, Iowa City, IA, USA; c MRC/University of Edinburgh Centre for Inflammation Research, Edinburgh, Scotland, UK

**Keywords:** Inflammation, Monocyte, Macrophage, Lipopolysaccharide, CFTR

## Abstract

**Background:**

We sought to address whether CF macrophages have a primary functional defect as a consequence of CFTR loss and thus contribute to the onset of infection and inflammation observed in CF lung disease.

**Methods:**

Monocyte derived macrophages (MDMs) were prepared from newborn CF and non-CF pigs*. CFTR* mRNA expression was quantified by rtPCR and anion channel function was determined using whole cell patch clamp analysis. IL8 and TNFα release from MDMs in response to lipopolysaccharide stimulation was measured by ELISA.

**Results:**

CFTR was expressed in MDMs by Q-rtPCR at a lower level than in epithelial cells. MDMs exhibited functional CFTR current at the cell membrane and this current was absent in CF MDMs. CF MDMs demonstrated an exaggerated response to lipopolysaccharide stimulation.

**Conclusions:**

In the absence of CFTR function, macrophages from newborn CF pigs exhibit an increased inflammatory response to a lipopolysaccharide challenge. This may contribute to the onset and progression of CF lung disease.

## 1 Introduction

Cystic fibrosis (CF) is an autosomal recessive, life limiting disease, associated with mutations in the gene that encodes Cystic Fibrosis Transmembrane Conductance Regulator (*CFTR*), a nucleotide and phosphorylation regulated anion channel in epithelia and other cell types [1]. CF is characterized by persistent and progressive lung infection and chronic inflammation leading to a decline in respiratory function and death. Whilst the functions of CFTR in airway epithelia are well established, there is also evidence of CFTR expression in myeloid cells and its absence has adverse consequences [2], [3], [4]. Defects in CF macrophage function and polarization have been reported [5], [6], [7], [8], but it is presently unclear whether these represent primary defects due to loss of CFTR function or an acquired response to chronic infection and inflammation.

To address whether CF macrophages have a primary functional defect as a consequence of CFTR loss, we utilized *CFTR*^*−/−*^ pigs (CF pigs) that have no lung inflammation at birth but exhibit pulmonary host defense defects and over time spontaneously develop lung disease with many similarities to CF in humans [9]. By studying newborn CF pig macrophages, we can investigate function before the onset of chronic inflammation, and thus assess the primary effects of loss of CFTR. Here we studied several properties of monocyte derived macrophages (MDMs) from newborn CF pigs and their wild type littermates (non-CF pigs) [10].

## 2 Materials and methods

### 2.1 Generation of MDMs

Peripheral blood mononuclear cells (PBMCs) were isolated from littermate newborn CF and non-CF pigs using Ficoll-Paque separation as described [11]. PBMCs were plated at 1 × 10^6^ per well in 48-well plates in Iscove's Modified Dulbecco's Medium (IMDM) for 1 h. Adherent cells were then differentiated in IMDM supplemented with 10% heat inactivated FBS, 1% penicillin/streptomycin, and 20 ng/ml of M-CSF (macrophage colony stimulating factor, rhM-CSF, PeproTech 300-25) for 6 days based on previously published work [12].

### 2.2 CFTR mRNA expression and function

To assess *CFTR* mRNA abundance in MDMs and airway epithelial cells, SYBR Green quantitative rtPCR was performed using CFTR primers and RPL4 primers. To measure CFTR ion channel activity in non-CF and CF MDMs, whole cell patch-clamp analyses were performed as previously reported [13].

### 2.3 MDM responses to a pro-inflammatory stimulus

MDMs were stimulated with 20 ng/ml or 200 ng/ml lipopolysaccharide (LPS, *E. coli*; Sigma L2630) for 24 h. Supernatants from stimulated cells were harvested and stored at − 80 °C until assayed. Porcine TNFα and IL8 were measured by ELISA, following the manufacturer's instructions (Duoset DY690B; DY535; R&D Systems).

## 3 Results

### 3.1 CFTR is expressed in porcine MDMs and is functional at the cell membrane

**Fig. 1:**
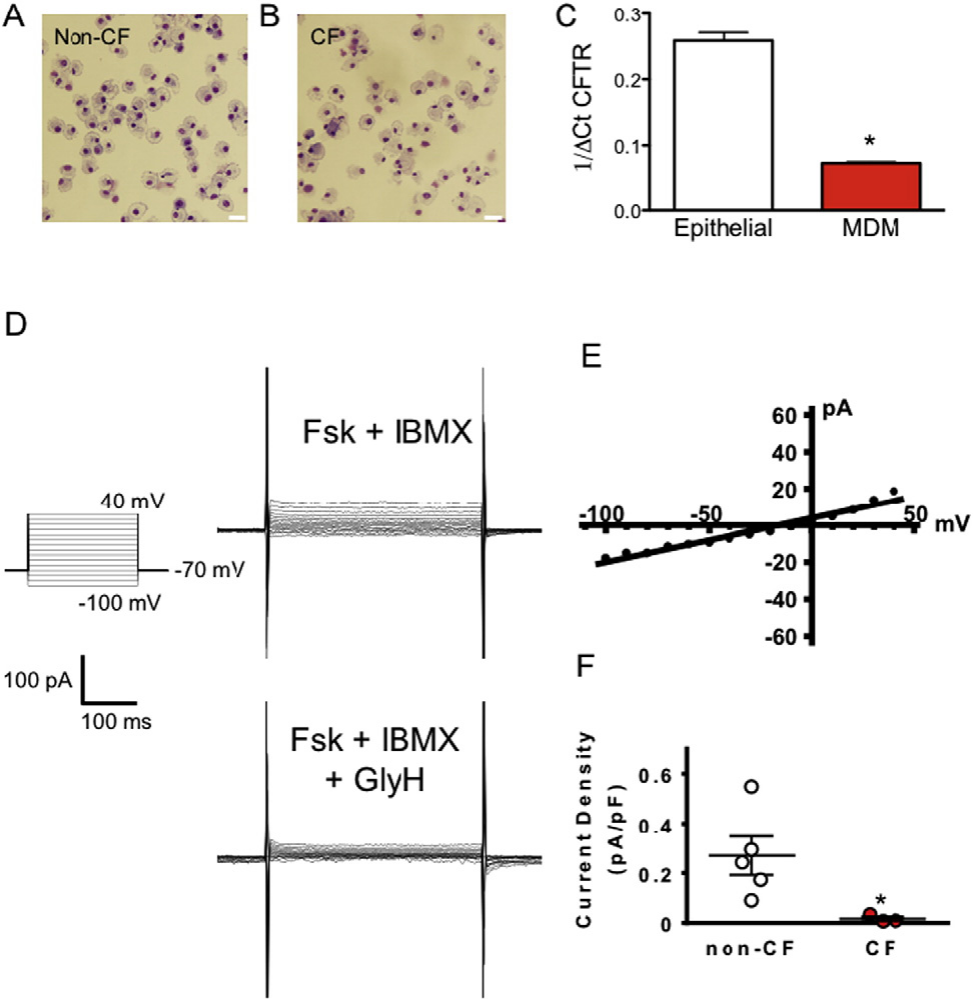
CFTR is expressed in monocyte derived macrophages. A, B. MDMs from non-CF and CF pigs have characteristic macrophage morphology and were phenotypically identical (scale bar = 20 μM). C. Quantitative rtPCR was carried out to assess *CFTR* mRNA abundance in non-CF porcine primary airway epithelia (*n* = 10) and MDMs (*n* = 7) (*P < 0.001, Mann–Whitney rank sum test). CFTR is functional in non-CF MDMs. D. Whole-cell current recorded in response to 10 μM forskolin (Fsk) and 100 μM 3-isobutyl-2-methylxanthine (IBMX, upper panel) and after adding 100 μM of CFTR inhibitor GlyH-101 (GlyH, lower panel) to the bath solution. Example of currents from one non-CF macrophage shown. Voltage-pulse protocol used for whole cell patch clamping is shown in inset on left. Current–voltage relationship of the GlyH-sensitive current is shown in E. F. GlyH-sensitive Fsk/IBMX-activated whole-cell current is absent in CF MDMs (holding potential = − 70 mV, *n* = 5 non-CF and 3 CF MDMs, *P < 0.05 Mann–Whitney rank sum test).

MDMs derived from non-CF and CF pigs exhibited characteristic morphology of macrophages on analysis of cytospin preparations (Fig. 1A and B). *CFTR*-specific amplicons were detected in both porcine airway epithelia and MDMs from non-CF animals and the fidelity of the PCR product was confirmed by Sanger sequencing (not shown). *CFTR* mRNA transcripts were detected in MDMs but at less abundance than in epithelial cells (Fig. 1C). We next used whole-cell patch-clamp recordings to detect CFTR activity. Whole cell currents from non-CF MDMs measured in response to forskolin and IBMX treatment demonstrated non voltage-activated Cl^−^ current that was inhibited by the CFTR inhibitor GlyH-101 (Fig. 1D), and showed an approximately linear current–voltage relationship characteristic of CFTR activity in this range (Fig. 1E). In contrast, similarly treated CF MDMs showed negligible current compared to non-CF cells, consistent with an absence of functional CFTR (Fig. 1F).

### 3.2 CF MDMs have enhanced cytokine production in response to LPS

**Fig. 2:**
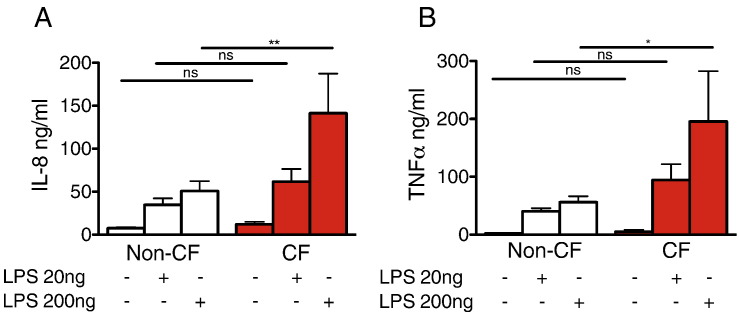
CF MDMs exhibit increased cytokine release in response to LPS stimulation. MDMs from newborn CF pigs release more IL8 (A) and TNFα (B) compared to non-CF in a dose-dependent manner (*n* = 8 non-CF and 8 CF, *P < 0.05, **P < 0.01, 1 way ANOVA with Newman–Keuls multiple comparison test).

Compared with non-CF controls, newborn CF MDMs released more IL8 and TNFα in response to LPS stimulation (Fig. 2A, B), and this was dose dependant.

## 4 Discussion

While the impact of loss of CFTR on airway epithelial cell function is well established, the effects on myeloid cells are more controversial. As reviewed by Bruscia [5], studies on monocytes, MDMs, and alveolar macrophages from humans [2], [7], [14] and animal models [6], [15] implicate loss of CFTR function in several phenotypes. These include enhanced responses to pro-inflammatory stimuli, impaired autophagy, altered cell death programs [14], [16], reduced endosomal acidification [15], compromised bacterial killing [7], and enhanced cytokine secretion at baseline [2], [17]. A possible confounding factor in human studies is the isolation of cells from subjects with established lung disease, raising the possibility that any differences from non-CF subjects may derive from the chronic inflammation related to CF lung disease rather than a primary effect of CFTR dysfunction. Since the macrophage is a key sentinel of innate immunity, we exploited features of the CF pig to address this issue. Newborn CF pigs have no pulmonary or systemic inflammation [10], but have resident airway host defense defects [9] and progress over time to develop lung disease with many similarities to humans with CF. We chose to study MDMs due to technical difficulties in harvesting adequate numbers of macrophages from the newborn CF pig lung, and acknowledge that there may be limitations in using derived macrophages rather than primary alveolar macrophages, in terms of observing their response to LPS [18]. Nevertheless, MDMs have been extensively employed in the literature to study macrophage function in several diseases and allow the comparison of our data from pigs with studies in humans with CF [14].

We found that CFTR is expressed in porcine MDMs and confers characteristic anion channel activity at the cell membrane. CF MDMs were morphologically indistinguishable from non-CF MDMs. MDMs from newborn CF pigs released more IL8 and TNFα in response to LPS, consistent with hyperresponsiveness to a TLR4 ligand [19]. Previous studies have reported exuberant production of cytokines by CF macrophages in response to LPS [6]. For the first time we show that this is a primary defect due to the absence of functional CFTR from macrophages, as it is observed in cells from newborn pigs that have no significant inflammatory lung disease. In humans, TNFα and IL8 levels show a positive correlation with CF disease progression and also induce chemotaxis of neutrophils to the respiratory tract [20], [21]. They are markers of inflammation that favor M1 macrophage polarization and thus could be a primary defect that contributes to the inflammatory process in the CF lung, a subject that has recently been extensively reviewed [22]. We speculate that this intrinsic defect in monocytes may be further augmented as the lung disease progresses, and may also result in abnormal communication with other arms of immunity. Interestingly, a study of newborn CF pig airway epithelia described blunted early inflammatory responses to a *S. aureus* challenge [23]. These differing responses to a pro-inflammatory stimulus in MDMs and airway epithelia from newborn CF pigs suggest that macrophage dysfunction could be a key driver of early lung disease in CF.

In summary, we demonstrate that CFTR function is absent in macrophages from CF pigs and this is associated with an increased inflammatory response to LPS challenge. Our findings support the presence of a primary defect in CF macrophages that promotes inflammation and offers a target for therapeutic development during the early stages of lung disease.

## Funding

This work was supported by NIH grants: P01 HL-51670, P01 HL-091842, K08HL097071, and the Roy J. Carver Charitable Trust, and the Cystic Fibrosis Foundation RDP. RDG was supported by a Wellcome Trust Fellowship (WT093767). We also acknowledge the support of the In Vitro Models and Cell Culture Core, partially supported by the Center for Gene Therapy for Cystic Fibrosis (NIH P30 DK-54759) and the Cystic Fibrosis Foundation.
